# Evaluation of halitosis in adult patients after treatment with photodynamic therapy associated with periodontal treatment: Protocol for a randomized, controlled, single-blinded trial with 3-month follow up

**DOI:** 10.1097/MD.0000000000016976

**Published:** 2019-09-27

**Authors:** Sergio dos Santos Romero, Tânia Oppido Schalch, Katia Llanos do Vale, Ellen Sayuri Ando, Marcia Pinto Alves Mayer, Joanna Paula Gaba Feniar, Kristianne Porta Santos Fernandes, Sandra Kalil Bussadori, Lara Jansiski Motta, Renata Matalon Negreiros, Anna Carolina Ratto Tempestini Horliana

**Affiliations:** a Postgraduate program in Biophotonics Applied to Health Sciences, University Nove de Julho, UNINOVE; b Department of Microbiology, Institute of Biomedical Sciences, University of São Paulo; c Assistant Professor of Specialization in Oral Maxillofacial Surgery and Traumatology at Fundecto – FFO, School of Dentistry – FOUSP, University of São Paulo, São Paulo, Brazil.

**Keywords:** periodontal disease, photodynamic therapy, randomized controlled clinical study

## Abstract

**Rationale:**

Halitosis is an unpleasant odor that emanates from the mouth. Studies show halitosis returns in a week, after treatment with PDT. Probably, bacteria living in the periodontal sulcus could recolonize the dorsum of the tongue. Until nowadays, there are no study in adult population that associates halitosis and periodontal treatment with follow-up evaluation. The aim of this randomized, controlled, single-blinded clinical trial is to treat oral halitosis in healthy adults with photodynamic therapy (PDT), associated with periodontal treatment and follow them up for 3 months.

**Patient concerns:**

the concerns assessments will be done over the study using anamnesis interviews and specific questionnaire.

**Diagnoses:**

halitosis will be evaluated by OralChroma.

**Interventions:**

The participants (n = 40) with halitosis will be randomized into 2 groups: G1-treatment with PDT (n = 20) or G2-cleaning of the tongue with a tongue scraper (n = 20).

**Outcomes:**

Halitosis will be evaluated by measuring volatile sulfur compounds using gas chromatography. After the treatments, a second evaluation will be performed, along with a microbiological analysis (RT-PCR) for the identification of the bacteria *T. denticola*. The assessment of halitosis and the microbiological analysis will be repeated. After that, patients will receive periodontal treatment. The participants will return after 1 week and 3 months for an additional evaluation. Quality of life will be measured by *Oral Health Impact Profile questionnaire* (OHIP-14).

**Lessons:**

This protocol will determine the effectiveness of phototherapy regarding the reduction of halitosis in adults. clinicaltrials.gov NCT 03996915.

**Ethics and dissemination:**

This protocol received approval from the Human Research Ethics Committee of *Universidade Nove de Julho* (certificate number: 3.257.104). The data will be published in a peer-reviewed periodical.

## 1 Introduction

Halitosis is known as an unpleasant odor from the mouth being a sign or symptom of imbalance.^[1,2]^ Its prevalence in the world is approximately 31.8%.^[3]^ The source of halitosis may be intraoral or extraoral.^[4]^ Halitosis of extraoral source originates in the upper airways or has a metabolic/systemic origin.^[2,5]^ Halitosis of intraoral source is the result of the degradation of organic substrates by anaerobic bacteria and the production of volatile sulfur compounds (VSC).^[5,6]^ Among the most important and common causes in the oral cavity are: lingual plaque (51%), periodontal disease (13%), or a combination of both (22%).^[2,3,7–9]^ The concentration of mucin in the saliva causes adhesion of the same to the lingual dorsum, especially in the region of the posterior third. In addition, desquamated epithelial cells from the buccal mucosa and proteolytic anaerobic microorganisms find 2 types of substrates: saliva proteins and proteins from desquamated epithelial cells. This set forms a whitish layer in this region of the tongue called tongue coating.^[9]^

A recent study evaluated the microbioma associated with halitosis. Among the most frequent bacteria we can mention *Treponema denticola*, *Porphyromonas gingivalis*, *Prevotella intermedia*, *Fusobacterium nucleatum* and *Campylobacter rectus*.^[10–13]^ These microorganisms release the VSCs which are products, resulting from the metabolism of these microorganisms. Among the main VSCs we can mention: hydrogen sulphide (SH_2_) - found on the back of the tongue, methylmercaptanes (CH_3_SH) - present in periodontal disease and dimethyl sulphide (CH_3_SCH_3_) of extraoral origin.^[14–17]^ The diagnosis of halitosis can be performed by gas chromatography, using an objective method, such as OralChroma (Abilit Corporation, Miyamae-KU Kawasaki-shi, Kanagawa, Japan).^[3,13,14]^

Treatment of halitosis can be done in several ways, including chemical reduction of microorganisms with mouthwashes (chlorhexidine 0.012%, essential oils and triclosan), mechanical reduction with a scraper or lingual brush and masking of the odor (chewing gum, spray and tablets).^[3]^ Recently, antimicrobial photodynamic therapy (PDT) has been studied as an alternative in the treatment of halitosis.^[18,19]^ PDT consists of the association of a photosensitizer (PS) that, by absorbing visible light at a suitable wavelength, releases reactive oxygen species.^[20]^ By absorbing energy, oxygen passes from the fundamental singlet state to the excited, short-lived singlet state. Once in this state, the PS may lose energy by nonradioactive, radioactive (fluorescence) processes or convert to the triplet state via intersystem intersection. From the triplet state (whose lifetime is longer compared to singlet), the PS can lose energy by nonradioactive, radioactive (phosphorescence) processes or participate in the formation of reactive oxygen species by electron transfer.^[21]^ This process is known as type I reaction or transfer of energy to molecular oxygen, generating singlet oxygen (type II reaction). This reaction generates energy, which is transferred to the oxygen molecules of the bacterium, leading to cell death.^[22]^

Although PDT is known as an immediate result treatment, 1 study^[8]^ shows that after 7 days, participants returned to baseline halitosis values, which underscores the statement that any treatment employed for halitosis should be accompanied with periodontal treatment. Also, the majority of halitosis studies focus in young adults, adolescents and children.^[8,23]^ There is a lack of halitosis study in older adults.

## 2 Methods

A single-center, randomized, controlled, single-blind clinical trial was designed in accordance with the criteria recommended for interventional trials in the SPIRIT statement. The protocol was approved by the Research Ethics Committee of Nove de Julho University (UNINOVE), number # 3.257.104 and registered at clinicaltrials.gov NCT 03996915. The study is in accordance with the *Declaration of Helsinki*. Patients who accept to participate will sign the Informed written consent form after verbal and written explanation of the study.

All data collected and laboratory samples will be identified and maintained confidential. Only the main investigators (authors of this article) will have complete access to the set of clean data.

The sample will comprise of 40 older adults’ patients (50 years or older) with halitosis. After agreeing to participate, patients will be evaluated in different moments: baseline- before halitosis treatments (T0), immediately after halitosis treatment (T1), after periodontal treatment (T2), after 1 week (T3) and three months later (T4). The clinical assessment and the PDT treatment will be performed by the same researcher, in all 4 moments at the Dental Clinic at Nove de Julho University – UNINOVE, São Paulo, Brazil, from September 2018 until March 2019. The measurements were performed by another researcher, who does not know the nature of treatments.

### 2.1 Sample size calculation

The size effect was calculated: 
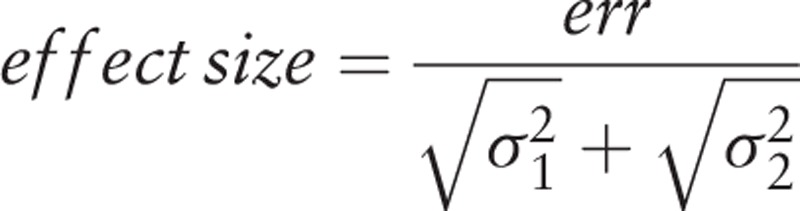


If the normal distribution hypothesis is rejected, the sample size should be corrected by approximately 5%. By observing statistical samples from the reference Mota et al, 2016^[8]^; to estimate the mean values and sample variance, we obtain the following sample sizes for each group:

### 2.2 Inclusion and exclusion criteria

Participants in this study will be evaluated to meet the eligibility criteria: both genders, with at least 10 teeth, PSR 0, 1 or 2, without any changes in the anatomy of the back of the tongue (geographical or fissured tongue) and positive halitosis (SH2 level higher than 112 ppb).

The following patients will be excluded: smokers or ex-smokers for less than 5 years and patients with hypersensitivity to the photosensitizer (PS).

### 2.3 Calibration of examiner

An experiment examiner will perform training exercises to maximize the reproducibility of measurements. Then, 10 individuals with positive halitosis will be evaluated using the OralChroma device. These individuals will be excluded of the study. The intraclass correlation coefficient (ICC) will be calculated, and an intra-examiner agreement (≥0.90) with regard to the halitosis must be ≥0.90.

### 2.4 Periodontal examination

An experienced and specialist investigator will use the *periodontal screening and recording* (PSR) system to assess the patients. PSR is a reliable, quick, and reproducible method for identifying patients, that may require a more complete evaluation of their periodontal health status. Using this system, the professional designates the patient's month in sextants and uses a probe with a 0.5 mm balled-end with a colored band extending from the 3.5 to the 5.5 mm área.^[24]^ Six sites around each tooth are probed. The code (0, 1, 2, 3, or 4) for charting is determined by how much of the colored band on a PSR probe is visible when it is inserted into the gingival sulcus: 0- colored area of probe remains completely visible in the deepest crevice in the sextante; no calculus or defective margins are detected; gingival tissues are healthy with no bleeding after gentle probing. 1- colored area of probe remains completely visible in the deepest probing depth in the sextant; no calculus or margins are detected; there is bleeding after gentle probing. 2- colored area of probe remains completely visible in the deepest probing depth in the sextante; supra- or subgingival calculus and/or defective margins are detected. 3- colored area of probe remains partly visible in the deepest probing depth in the sextant. 4- colored area of probe completely disappears, indicating probing depth of greater than 5.5 mm. Each sextant is assigned a code based on the highest probing value obtained on any tooth in that sextant.^[24,25]^ Subjects having 1 or more sextants code 3 or 4 require a complete evaluation of periodontal status, with specific treatment and will not be included in this study.

### 2.5 Randomization

An external researcher who will not participate in this study will perform randomization through the Microsoft Excel program, version 2017. The same researcher will randomize into 5 blocks with 4 patients (1:1) and groups will be designated as A or B. The letters (A or B), drawn will be placed into opaque envelopes labeled with sequential numbers. The envelopes will be sealed and remain in the same numerical order in a safe place until treatments. This data will only be revealed after statistical analysis. As the study will be performed in a single-blinded manner, the operator will not know which kind of treatment will be performed. The patient will not be blinded because the nature of treatments (PDT and tongue scrapers).

### 2.6 Design

This is a controlled, randomized, single-blind clinical trial:

G1 – (experimental group -PDT) 20 patients with halitosis, 1 session of PDT will be performed with the PS. PS will be applied in enough quantity to cover the middle third and back of the tongue and wait for 5 minutes. The irradiation will be performed with low intensity laser λ = 660 nm, in 6 points, 9 J per point and radiant exposure of 318 J/cm^2^.
G2 – (positive control group - tongue scrapper) 20 patients with halitosis treatment tongue scraping will be performed by the same operator. Posterior-anterior movements will be performed with the scraper over the lingual dorsum in order to promote the mechanical removal of tongue coating.

### 2.7 Methodology for irradiation – low level laser specifications and dosimetry

**Figure 1:**
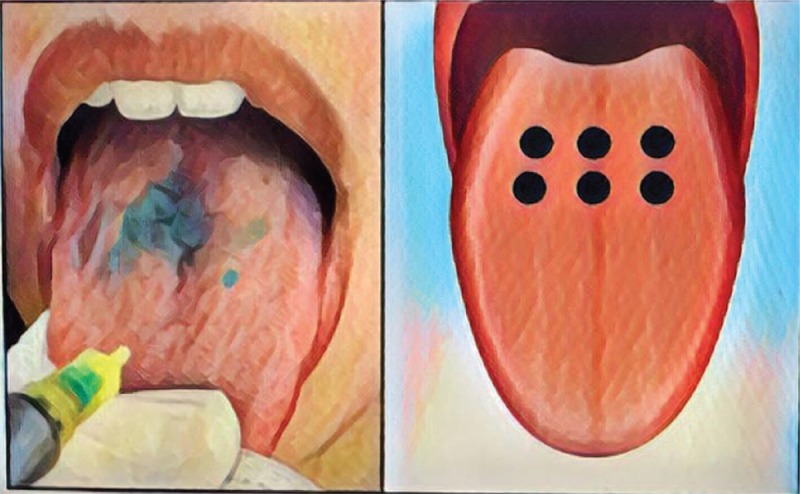
Photodynamic therapy- Photosensitizer and irradiation points of laser.

All participants of G1 group will be submitted to the application of 0.005% methylene blue (Chimiolux 5, DMC, São Carlos, São Paulo) in enough quantity to cover the middle and posterior third of the of the dorsum of the tongue with a pre-irradiation time of 3 minutes. Will be performed irradiations with the red laser diode (λ = 660 nm) with output power of 100 mW, 9 J, 318 J/cm^2^ and irradiance of 3537 mW/cm^2^ with a point application method and in direct contact with the tongue (Therapy XT, DMC, São Carlos, SP, Brazil).^[18,19]^ The irradiations will be done in 6 points with a distance of 1 cm between the points, considering the scattering halo and the effectiveness of PDT (Fig. 1). To completely remove the dye, the tongue will be washed in abundance with saline solution. During the laser application both patient and operator will wear goggles.

### 2.8 Treatment with tongue scraper

All participants of G2 group will receive treatment with tongue scraper as described below: the lingual scraper will be positioned on the posterior dorsum of the tongue, in the region of the valved papillae, and will pull with slight pressure to the apex of the tongue, obeying the manufacturer's instructions. This procedure will only be performed once.^[26]^

### 2.9 Periodontal treatment and oral hygiene guidance

After the treatment on the back of the tongue a new evaluation of VSC by gas chromatography (OralChroma, Abilit, Japan) and new collection for microbiological analysis of all patients of both groups will be performed.

Periodontal treatment will be performed, removing in a single session, all biofilm and calculus with universal curettes (Hu-Friedy, Chicago, IL) and ultrasonic device (EMS-Piezon Systems PM200, Nyon, Switzerland), by an experienced and specialist investigator. All participants will receive oral hygiene guidance (HBO). After periodontal treatment of these patients, the VSC will be evaluated and the microbiological collection will be repeated.

### 2.10 Outcome measures

#### 2.10.1 Halitosis measurement (gas chromatography test)

**Figure 2:**
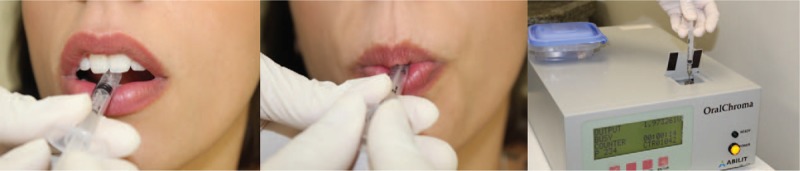
Oral Chroma Device (Abilit, Japan), VCS results.

The portable Oral Chroma (Abilit, Japan), a sensitive semiconductor gas sensor, will be used for the assessment of halitosis. Oral air collection will follow the manufactured guide lines (Oral Chroma Manual Instruction). A syringe will be placed in the patient's mouth with the plunger completely inserted and the participant will breathe through the nose with the mouth closed for 1 minute. The plunger will then be withdrawn to fill the chamber with air. The gas injection needle will be placed on the syringe and the plunger will be adjusted to 0.5 ml. This air will be injected into the input of the device in a single motion (Fig. 2). There is a software that produces a graph and stores at the device the values of concentration of VSCs (from 0 to 1000 ppb). Values above 112 ppb are indicators of halitosis. This procedure will be performed always in the morning and participants will receive a list of products to be avoided or forbidden, as spices, alcohol, coffee and chewing gums. The halitosis measurement will be done several times: before, after PDT or scraper, after periodontal treatment, after 1 week and 3 months later.

#### 2.10.2 Microbiological analysis

Sampling will be performed on the back of the tongue before and after halitosis treatment, after 1 week and 3 months later. Samples of the tongue coating will be collected with swab, placed in sterile identified microtubes (containing Tris-EDTA) and stored at -80oC until analyzed. After thawing, the samples will be vortexed for one minute. DNA extraction will be performed with the Master Pure DNA Extraction Kit (Epicenter Technologies Corp., Chicago, IL) according to the manufacturer's instructions. The purified DNA will be resuspended in TE buffer. The quantitative analysis will be performed using the Step One Plus Real-Time PCR System (Applied Biosystem, Foster City, CA) and the products detected by fluorescence using the Quantimix Easy SYG Kit (Biotools, Madrid, Spain) by the manufacturer. For the reaction, 10 μl of SYBR Green, 0.5 μl DNA template, 200 mM of each primer corresponding to the genes specific for identification of *T. denticola*, will be used.^[27]^

#### 2.10.3 OHIP

*Analysis of Oral Health Impact Profile* (OHIP-14 questionnaire): The OHIP-14 is an instrument that consists of 14 items, arranged in 7 domains among the following subscales: functional limitation, pain, psychological discomfort, physical disability, psychological disability, social disability and social handicap. The answers are given corresponding to a total of 5 points on a Likert-type scale. The scale included the following responses: never (coded 0), hardly ever (coded 1), occasionally (coded 2), fairly often (coded 3), and very often (coded 4). The OHIP-14 scale ranged from 0 to 56, with higher scores indicating poorer QoL.^[28]^ Oral health related to quality of life (OHRQoL), will be assessed by Brazilian version of OHIP-14 questionnaire.^[29]^ A calibrated examiner will apply the OHIP-14 on the day that the patient history is taken, (baseline) and 3 months after the treatment for halitosis. The items of the OHIP-14 are shown in Table 1.

**Table 1:**
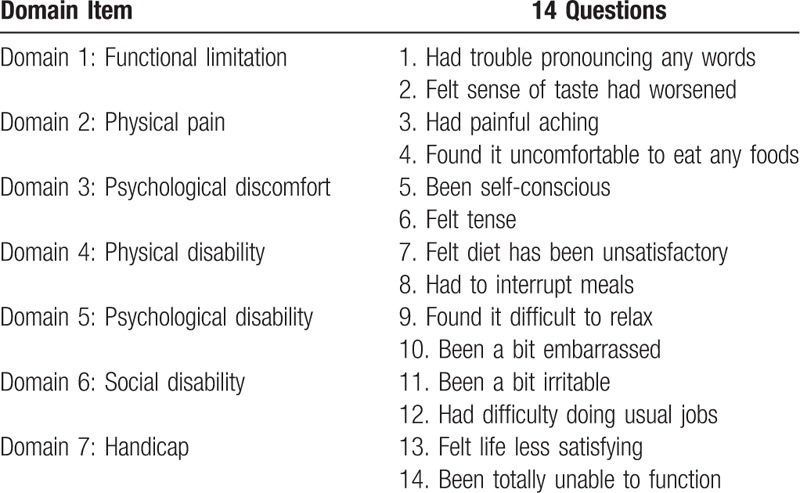
Ohip-14 questionnaire and its domains.^[28]^.

**Figure 3:**
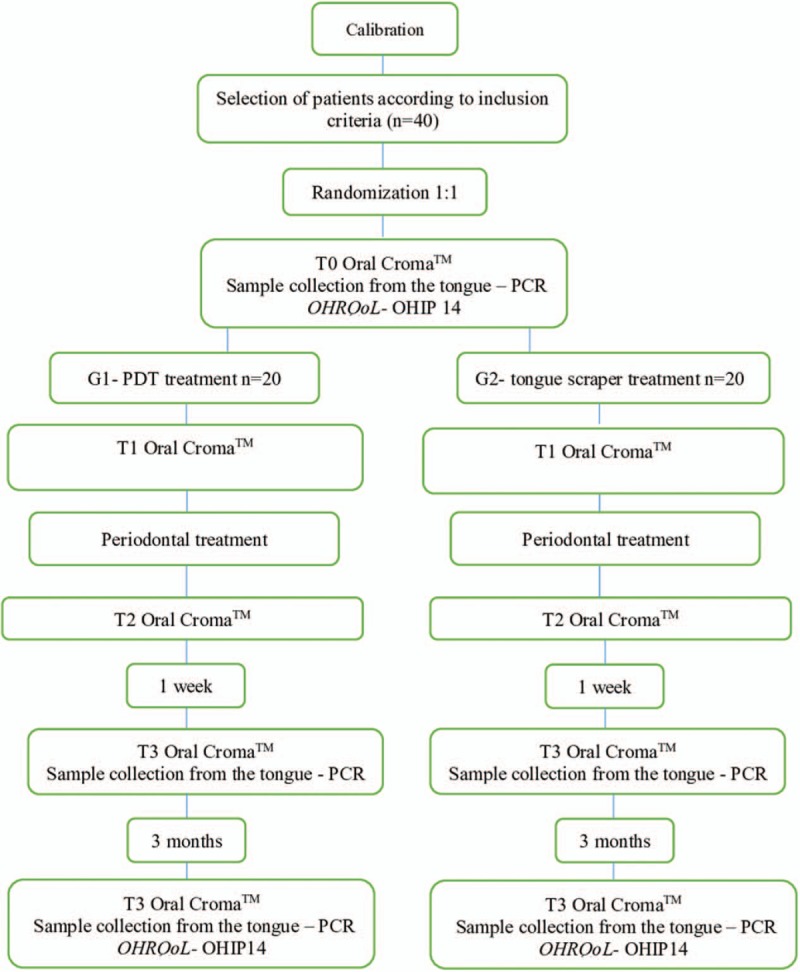
Flowchart presenting an overview of the study.

A flowchart is presented for an overview of the study (Fig. 3).

### 2.11 Statistical analysis

The normality of the data will be determined by the Lilliefors test. If normality is demonstrated, analysis of variance (*t* test Student, Bioestat 5.3, Pará, Brazil) will be used to compare continuous and dependent variables among the groups (G1 and G2). Mann–Whitney test will be used if non-normal distribution is demonstrated. The data collected at 1 week evaluation will be contrasted to the baseline data. A *P* value < .05 will be considered indicative of statistical significance. The *x*^2^ test will be used to compare categorical variables among G1 and G2, at baseline and the 1 week evaluation. These data will be expressed as mean ± standard deviation.

## 3 Discussion

PDT for the treatment of halitosis is known by causing immediate effects results in an unpleasant odor that emanating from the mouth.^[18]^ However, one study shows halitosis returns in a week after treatment with PDT.^[8]^

Probably, bacteria residing in the periodontal sulcus could recolonize the dorsum of the tongue, therefore we design this study promoting a complete mouth cleaning at once, in order to verify 1 week and 3-month follow-up. We think that immediate effects of PDT should be prolonged by complete one session decontamination of the mouth, by periodontal treatment.

Treatment of halitosis can be done in several ways, including chemical reduction of microorganisms with mouthwashes (chlorhexidine 0.012%, essential oils and triclosan), mechanical reduction with a tongue scraper, masking of the odor (chewing gum, spray, and tablets).^[3]^ However, these treatments may present disadvantages, such as excessive excoriation of the tongue surface with transudation and desquamation, worsening halitosis and causing discomfort when eating acid or bitter foods.^[8]^

PDT has been shown good results in treating halitosis,^[18,19]^ there are no reports of adverse effects or toxicity.^[20,22,30,31]^ As control, will be used a comparative group that will be treated with lingual scraper, the most conventional therapy in the treatment of halitosis.^[8]^

Patients with changes in the anatomy of the back of the tongue (geographical or fissured tongue) will not be included in the study. The fissures and modified papilla may favor the bacterial accumulation predisposing halitosis.^[32]^ Smokers and newly ex-smokers will be excluded, because the air exhaled by them can be mistaken for halitosis.^[33]^ Participants will be randomized in 1:1 equivalence group because both treatments are effective, and we do not expect adverse effects. Also, we decide to block the sample in 5 blocks of 4 patients, because of equivalence in the number of patients in both groups in case of we decide to include 1 more group. We expect a higher drop out because halitosis is a slight health problem and we expect some difficult to convince them to realize 2 follow up. When we achieve 8 patients per group, we will decide for a third treatment group.

It is not possible that this study becomes double-blind study, because patient will always know which therapy is being performed, since lingual scraper and PDT are completely different treatments. However, we planned a blind operator (who will collect OHIP questionnaires, microbiological analyses and VSC by gas chromatography) because he will not know the nature of treatments.

The diagnosis of halitosis will be done using OralChroma, since it is an effective and objective method which is possible to measure the halitosis intensity based on gas chromatography.^[3,14,15]^

Halitosis has also been linked to the presence and severity of periodontal diseases. Studies have shown that concentrations of VCS increase (mainly methyl mercaptan) in patients with periodontal disease, (probing > 4 mm), but it has not been studied to date if periodontal treatment is able to reduce the halitosis of these patients.^[6,16,17]^ Based on this information, in this study, patients who present halitosis even after PDT or tongue scraper treatment will undergo periodontal treatment in our study.

Microbiological analysis will be another outcome measure in this study. The presence of *Treponema denticola* will be assessed since recent studies show that these species are very prevalent in cases of halitosis.^[10–13]^ An accepted theory is that aspiration of oral bacteria could cause or exacerbate systemic disease.^[34–37]^ Based on this theory, it is important to reduce bacterial load.

A valid measure of the halitosis impact on quality of life, is required for the assessment of treatment effectiveness. The HALT (halitosis associated life quality test) questionary is also very useful for reliable results.^[38]^ This tool can immensely help monitoring one's treatment progress, but we still do not have a version for Brazil, that is the reason why we chose OHIP.

The specifications and dosimetry of laser in this search will be the same used in studies that used PDT to treat halitosis and obtained good results. Irradiations with the red laser diode (λ = 660 nm) with output power of 100 mW, 9 J, 320 J/cm^2^ and irradiance of 3537 mW/cm^2^ with a point application method and in direct contact with the tongue, 6 points with a distance of 1 cm between the points, showed significantly reduce of the halitosis levels.^[18,19,8]^

We expect that this randomized, single blinded, controlled clinical trial will be able to treat oral halitosis in healthy adults.

## Author contributions

**Validation:** Anna Carolina Ratto Tempestini Horliana, Lara Jansiski Motta

**Conceptualization:** Sergio dos Santos Romero, Anna Carolina Ratto Horliana.

**Data curation:** Kristianne Porta Fernandes, Sandra Kalil Bussadori, Lara Jansiski Motta.

**Formal analysis:** Sergio dos Santos Romero, Katia Llanos do Vale, Marcia Alves Mayer.

**Investigation:** Sergio dos Santos Romero, Tania Oppido Schalch, Renata Matalon Negreiros.

**Methodology:** Marcia Alves Mayer, Kristianne Porta Fernandes, Anna Carolina Ratto Horliana.

**Project administration:** Renata Matalon Negreiros, Anna Carolina Ratto Horliana.

**Resources:** Sergio dos Santos Romero, Katia Llanos do Vale, Ellen Sayuri ando.

**Supervision:** Sandra Kalil bussadori.

**Validation:** Lara Jansiski Motta.

**Writing – original draft:** Sergio dos Santos Romero, Tania Oppido Schalch, Joanna Gaba Feniar, Renata Matalon Negreiros.

**Writing – review & editing:** Tania Oppido Schalch, Joanna Gaba Feniar, Sandra Kalil Bussadori, Renata Matalon Negreiros, Anna Carolina Ratto Horliana.
